# Vesicle Motion during Sustained Exocytosis in Chromaffin Cells: Numerical Model Based on Amperometric Measurements

**DOI:** 10.1371/journal.pone.0144045

**Published:** 2015-12-16

**Authors:** Daungruthai Jarukanont, Imelda Bonifas Arredondo, Ricardo Femat, Martin E. Garcia

**Affiliations:** 1 Institute of Physics and Center for Interdisciplinary Nanostructure Science and Technology (CINSaT), Universität Kassel, Kassel, Germany; 2 División de Matemáticas Aplicadas, IPICYT, Camino a la Presa San José 2055, Lomas 4^a^ Sección., San Luis Potosí, México; UPR 3212 CNRS -Université de Strasbourg, FRANCE

## Abstract

Chromaffin cells release catecholamines by exocytosis, a process that includes vesicle docking, priming and fusion. Although all these steps have been intensively studied, some aspects of their mechanisms, particularly those regarding vesicle transport to the active sites situated at the membrane, are still unclear. In this work, we show that it is possible to extract information on vesicle motion in Chromaffin cells from the combination of Langevin simulations and amperometric measurements. We developed a numerical model based on Langevin simulations of vesicle motion towards the cell membrane and on the statistical analysis of vesicle arrival times. We also performed amperometric experiments in bovine-adrenal Chromaffin cells under Ba^2+^ stimulation to capture neurotransmitter releases during sustained exocytosis. In the sustained phase, each amperometric peak can be related to a single release from a new vesicle arriving at the active site. The amperometric signal can then be mapped into a spike-series of release events. We normalized the spike-series resulting from the current peaks using a time-rescaling transformation, thus making signals coming from different cells comparable. We discuss why the obtained spike-series may contain information about the motion of all vesicles leading to release of catecholamines. We show that the release statistics in our experiments considerably deviate from Poisson processes. Moreover, the interspike-time probability is reasonably well described by two-parameter gamma distributions. In order to interpret this result we computed the vesicles’ arrival statistics from our Langevin simulations. As expected, assuming purely diffusive vesicle motion we obtain Poisson statistics. However, if we assume that all vesicles are guided toward the membrane by an attractive harmonic potential, simulations also lead to gamma distributions of the interspike-time probability, in remarkably good agreement with experiment. We also show that including the fusion-time statistics in our model does not produce any significant changes on the results. These findings indicate that the motion of the whole ensemble of vesicles towards the membrane is directed and reflected in the amperometric signals. Our results confirm the conclusions of previous imaging studies performed on single vesicles that vesicles’ motion underneath plasma membranes is not purely random, but biased towards the membrane.

## 1 Introduction

Regulated exocytosis, i.e., vesicle-mediated release of neurotransmitters from inside the cell to its environment, is a fundamental process in bio-signaling [1–4]. Failure in exocytosis is associated with numerous severe conditions like cancer, Down syndrome and Alzheimer’s [5–7].

In chromaffin cells, a common pathway of regulated exocytosis [1, 3, 8] consists in the following sequential processes: (i) Catecholamines-filled vesicles are transported from the cell interior towards the cell periphery. (ii) Vesicles are physically connected to the membrane (docked) at special areas called active sites. These sites are composed of unique proteins that make vesicle docking possible [8]. (iii) After docking, vesicles are brought to a ready-releasable state by an ATP-dependent process called priming. (iv) Immediately upon a rise in cationic stimulation, primed vesicles release catecholamines to the extracellular space through membrane-fusion.

Exocytosis involves a complex molecular machinery, consisting of different proteins like SNAREs (soluble N-ethylmaleimide sensitive factor attachment protein receptors) synaptotagmins, complexins and Sec1/Munc18, which play important roles in the accomplishment of docking, priming and membrane-fusion [9–18]. The last step of membrane-fusion is triggered by the increase of intracellular [Ca^2+^]_*c*_ which is also essential for regulating priming and docking [19–21] (for more detailed documentations on exocytosis, see also refs. [2–4, 8]).

The above generic description is based on comprehensive biochemical and biophysical investigations from the last years [1, 11, 15, 16, 21–35]. They include molecular manipulations [11, 15, 16, 21, 25], electrophysiological techniques [26–28] and optical observations [29–33]. Currently, primed vesicles’ movement, the priming molecular machinery, membrane-fusion and pore formation are largely understood. However, details of vesicle transport to the active sites of the membrane are still unclear.

As mentioned before, docking describes the state in which vesicles are physically connected to the plasma membrane by a set of proteins [14]. In the absence of a stimulus, docked vesicles can be characterized by means of electron microscopy [36, 37], as those are located next to the membrane (within a distance of ≤30 nm). In this case, however, also primed vesicles would be included in this definition, since from electron microscopy images it is not possible to distinguish between primed and docked vesicles. Other techniques like total internal reflection fluorescence (TIRF) microscopy can distinguish primed and docked vesicles by their different mobilities (primed vesicles are almost immobile) [29, 30, 32, 33]. It is important to point out that most vesicles are initially non-docked and reside deep inside the cell in the reserve pool.

When exocytosis starts as a response to a stimulus, the pools of already docked and primed vesicles are fully or partially depleted or at least considerably reduced, within 1–10 s [4]. Then, the so called sustained-release regime is reached, in which docking of new vesicles at the plasma membrane followed by priming and membrane-fusion occurs. This means that the sustained phase is the stationary regime in which the contribution from already primed and docked vesicles is small. this regime, one can clearly distinguish two distinct time scales: ‘slow’ transport of vesicles towards the membrane and ‘fast’ docking, priming and membrane-fusion. Evidence of this ‘fast’ fusion reaction was the observation of ‘crash fusion’ reported in chromaffin cells, in which catecholamines are rapidly released without stable docking and priming [36, 38], as well as in synapses [33, 39, 40], islet *β* cells [41, 42], natural killer cells [43], and other cell types [14]. About 22% of the vesicles undergo ‘crash fusion’[38] The time scale for docking and priming in the sustained phase was reported to be within 50 − 300 ms in chromaffin cells [38], and within ∼100 − 200 ms in calyx of held [39, 44] and other cells [21, 45]. One of the reasons for rapid release may be a requirement of fewer SNARE complexes during the sustained phase [11, 46]. More support for fast fusion is presented in Refs. [21, 47, 48].

Thus, all the facts mentioned above indicate that there is a clear separation of time scales in the sustained regime. The slow mechanism of vesicle transport would therefore determine the exocytosis rate, especially in neuroendocrine cells, where vesicles do not recycle [4]. Therefore, we expect that the dynamics of release events during the sustained exocytosis, if the docked pool is completely depleted, may strongly depends on the transport of vesicles towards the membrane. Hence, the rate of electrophysiologically detected fusion events should reflect the dynamics of vesicle motion. Note, however, that the releases from the not completely depleted docked pool might also be present in the measured signals.

The existing models for vesicle movement toward the active sites are based on real-time observations of vesicle trajectories underneath the membrane using TIRFM [29, 30, 49], and 3D map confocal imaging technique [31]. Careful investigations on chromaffin cells [29, 30, 49] as well as other neurosecretory systems [50] lead to the conclusion that vesicles approach the active sites following a directed motion (active transport) preceding membrane fusion. However, the conclusions drawn from such visualization studies are rather restricted due to the limitation in resolution and penetration depth [51]. Typical observation depths of TIRFM are only in the range of ∼100 − 200 nm, and the information regarding motion in the *z* direction is only indirect. Moreover, the number of fusions detected by TIRFM is usually one or two orders of magnitude below amperometry [28, 36, 52].

In this work, we developed a model for vesicle transport and arrival at the cell membrane in order to simulate and analyze the release statistics of an ensemble of vesicles participating in exocytosis. For comparison, we performed high stimulus *ex-vivo* amperometric experiments on bovine chromaffin cells. Amperometric measurements allow us to identify quantal release of single vesicles from the time series of current-spikes [26, 53, 54]. Cells were stimulated with high concentration of Ba^2+^ and not with Ca^2+^. This has the following advantages for our study: (i) Ba^2+^ induces prolonged sustained releases, since it favors a more stable level of intracellular concentration of Ca^2+^ than direct calcium stimulation [55–57], and (ii) Ba^2+^ was shown to specifically stimulate the reserve pool of vesicles residing originally deep inside the cell [58]. However, we point out that there is so far no clear evidence that results obtained by Ba^2+^ application provide a one-to-one correspondence with those occurring during sustained release under physiological conditions. In addition, we performed a careful analysis of data to extract information on the release events from the measured signals and to complete a statistical study. In particular, we stress here the importance of using time rescaling schemes to make statistical averaging over isolated cells comparable, since individual isolated cells may exhibit different exocytosis rates and lifetimes. Our results indicate that the probability distribution for exocytosis events deviates from that of a Poisson process and rather follows a two-parameter gamma distribution. This result indicates that release occurrence is not random.

In order to understand this behavior we applied our numerical model based on Langevin simulations to interpret the experiments. First, we used a simple diffusive model by considering vesicles in the cell as confined Brownian particles. In this purely diffusive model simulations suggest Poisson statistics which confirms the connection between Brownian motion and random spike statistics. We were able achieve a good agreement between simulations and amperometric experiments by assuming that vesicles stochastically move under an attractive harmonic potential towards the membrane, which yields a gamma distribution of interspike times. Moreover, in order to provide a more realistic description, we also included the statistics of the priming/fusion reaction times in our model. We were able to demonstrate that fusion-prime/fusion processes do not modify the distributions of interspike-times. Therefore, we conclude that vesicle transport is responsible for the release distributions. Our results confirm the active transport of vesicles toward the membrane as suggested by TIRFM and other optical studies.

## 2 Experimental method

### 2.1 Cell Culture and Preparation

We prepared bovine chromaffin cells by collagenase digestion of the medulla of adrenal glands obtained from a local slaughterhouse (Meaux, France, coordinate: 48°57′37′′N 2°53′18′′E). Cells were purified and cultured using previously described methods [59] for 3–10 days. The purified chromaffin cells were plated on poly-L-lysine coated glass coverslips, and then placed into 24-well plates at a density of 4 x 104 cells/ cm^2^. The cells were incubated in a CO_2_ atmosphere (5%) at 37°C and used within 24 hours.

### 2.2 Electrode Preparation and Single Cell Experiments

Carbon fiber microelectrodes (7-mm diameter, Thornel Carbon Fibers, Cytec Engineered Materials, Greenville, SC, USA) were constructed as described in Refs. [60, 61]. Electrode tips were polished (45°) on a diamond dust-embedded micropipette beveling wheel (Model EG-4, Narishige Co., Tokyo, Japan) for 5–10 min. before experiments. Only electrodes with a very stable amperometric baseline current were used for cell measurements. Cells were prepared by placing each coverslip into a plastic dish (35 mm) filled with isotonic physiological saline (154 mm NaCl, 4.2 mm KCl, 0.7 mm MgCl_2_, 11.2 mm glucose, 10 mm HEPES, pH 7.4, 5 mL). After positioning the dish onto the stage of an inverted microscope (Axiovert-135, Carl Zeiss, Germany), the carbon fiber microelectrode surface was positioned with a micromanipulator (Model MHW-103, Narishige Co., Tokyo, Japan) at submicrometric distance from the membrane of an isolated chromaffin cell. The close proximity of the electrode surface to the cell surface was often seen by a slight deformation in the outline of the cell. Then, a glass microcapillary (20–30 *μ*m diameter) was positioned with a second micromanipulator near the cell (20–30 *μ*m) and used (Femtojet injector, Eppendorf Inc., Hamburg, Germany) to inject a stimulating solution towards the cell surface. We stimulated exocytosis with a Ba^2+^ solution composed of 2 mm BaCl_2_ in Locke buffer without carbonates supplemented with 0.7 mm MgCl_2_ for 10 s. The micro-electrode was kept in place during the stimulations and all along the secretion processes which take 5–10 minutes. Each cell was only stimulated once. All experiments were performed at room temperature. Electrodes were held at +0.65 V with respect to a silver/silver chloride reference electrode using a modified picoamperometer (model AMU130, Radiometer Analytical Instruments, Copenhagen, DK), for which the adjustable time-response was set at 50 *μ*s. The output was digitalized at 40 kHz, displayed in real time and stored on a computer with no subsequent digital filtering.

## 3 Amperometric data processing

As mentioned before, one expects the essential information of vesicle movement towards the active sites at the membrane during sustained exocytosis to be contained in the statistics of the release events (spike sequence). Our experimental setup was designed to have prolonged high releases (∼900 spikes). In this section we present a method to transform our amperometric traces into uniform spike sequences.

### 3.1 Identification of exocytotic events

**Fig 1:**
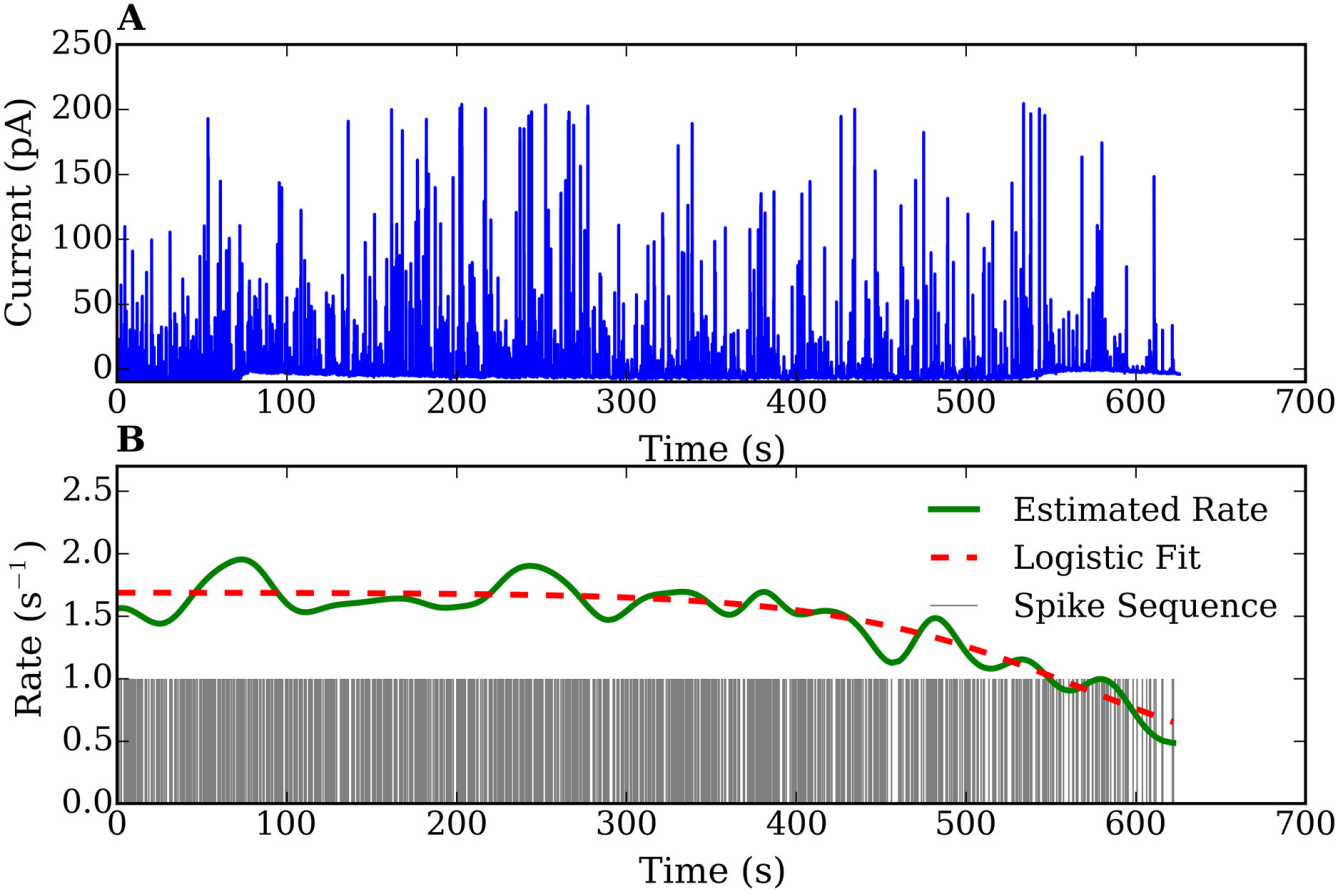
(A) An amperometric trace recorded from a chromaffin cell. (B) Extracted spike sequence and continuous curve showing the estimated rate using the density function method. The dashed curve shows a fit of the rate using a logistic function.

A quantal release of a single vesicle can be identified as an amperometric peak. The continuous amperometric measurement is transformed into a spike sequence (i.e., point process data) of the form {*t*_0_, *t*_1_, …*T*} on the interval (0, *T*], which is an appropriate object for our statistical analysis. To extract the release events, we visually and computationally inspected the measured amperometric traces. The systematic steps followed to obtain and process data are as the following. We first prepared the raw traces for spike detection by smoothing the signals and removing the baseline currents. Then, we used a spike-detection algorithm to extract the exocytosis events sequence. Note that we were only interested in the time between events and not in the particular shape of the single peaks. The obtained signals were considered as exocytotic peaks when their maximal currents were 3 times higher than the root mean square noise (0.2 to 0.5 pA) of the baseline current [53]. Special attention was paid to extract only spikes with shapes corresponding to full release events, e.g. spikes with specific rising and decaying characteristics. Generally, 500 to 900 peaks were isolated from each trace following this criterion (see, for instance, Fig 1(A). Extracted exocytosis events result in a series of spikes in Fig 1(B)). For a detailed explanation see S1 File.

### 3.2 Exocytosis-rate calculation

One can characterize the intensity of exocytosis events as a function of time by defining a rate function (intensity function), which characterizes the probability distribution of the spike sequence and is required in the probabilistic model prediction. A spike rate λ(*t*) is defined as the probability that a spike occurs within a small interval, [*t*, *t* + *Δt*]. A widely used method to determine λ(*t*) is the so called ‘Peristimulus time histogram’ (PSTH) [62, 63]. The idea consists in simply counting the number of spikes and dividing it by the time interval *Δt*. However this method does not yield smooth rates as a function of time, and therefore we introduce in this work the ‘density function method’.

#### Density function method

In order to obtain a smooth function λ(*t*), we replaced the *n* ^*th*^ spike as a normalized density function *ω*(*t* − *t*_*n*_) having a maximum at *t*_*n*_, and vanishing as |*t* − *t*_*n*_| grows. A good approximation for *ω*(*t* − *t*_*n*_) can be a Gaussian. Then, the spike rate is given by

$$\begin{array}{c} \lambda \left(t\right)=\sum_{n=1}^{N}\omega \left(t-t_{n}\right), \end{array}$$ (1)

with the normalization condition $\int_{-\infty }^{\infty }dt\;\omega (t-t_{n})=1$. *N* is the number of spikes in the interval [*t*, *t* + *Δt*].

We show in Fig 1 both the amperometric signal obtained from a measurement as well as the spike sequence and the spike rate calculated using the density function method. In the illustrated measurement, the sustained exocytosis was active for about 10 min, producing ∼900 release events. Since the number of spikes is sufficiently large, a single spike sequence is appropriate for an accurate statistical analysis. The estimated rate function stays approximately constant for about 7 min and then shows a decay, which is characteristic for *ex-vivo* experiments. No short bursting right after the stimulation is observed, indicating a well-behaved sustained regime. The estimated rate function of other 28 experimental signals show similar trends (temporary plateau and decaying behavior) although they have different averages and durations (data not shown).

### 3.3 Time-rescaling transformation

*Ex-vivo* experiments on isolated cells have the drawback that cell activity decays in time. The rate of exocytosis varies from cell to cell. This is problematic for the statistical analysis and for the interpretation of statistical measures averaged over an ensemble. In order to overcome this problem we applied a time-rescaling transformation which maps spike sequences with different time varying rates into spike sequences of constant rates. This makes signals from different cells comparable and also allows one to classify the spike sequences by referring to stationary point processes. The method described below is based on the time-rescaling theorem [64, 65].

We consider a spike sequence {*t*_*i*_} on the interval (0, *T*] with time-dependent rate λ(*t*), where λ(*t*)≥0, ∀*t*. The cumulative intensity function is given by the integrated rate,

$$\begin{array}{c} \Lambda \left(t\right)=\int_{0}^{t}\lambda \left(s\right)ds. \end{array}$$ (2)

This integral is known as the time-rescaling transform [64, 65]. Λ(*t*) rescales the original spike times into independent and identically distributed random spikes. One denotes *τ* as the rescaled time Λ(*t*), and *u*_*i*_ refers to the interspike times of the rescaled processes, which are given by

$$\begin{array}{c} u_{i}=\Lambda_{t_{i-1}}\left(t_{i}\right)=\int_{t_{i-1}}^{t_{i}}\lambda \left(s\right)ds \end{array}$$ (3)

**Fig 2:**
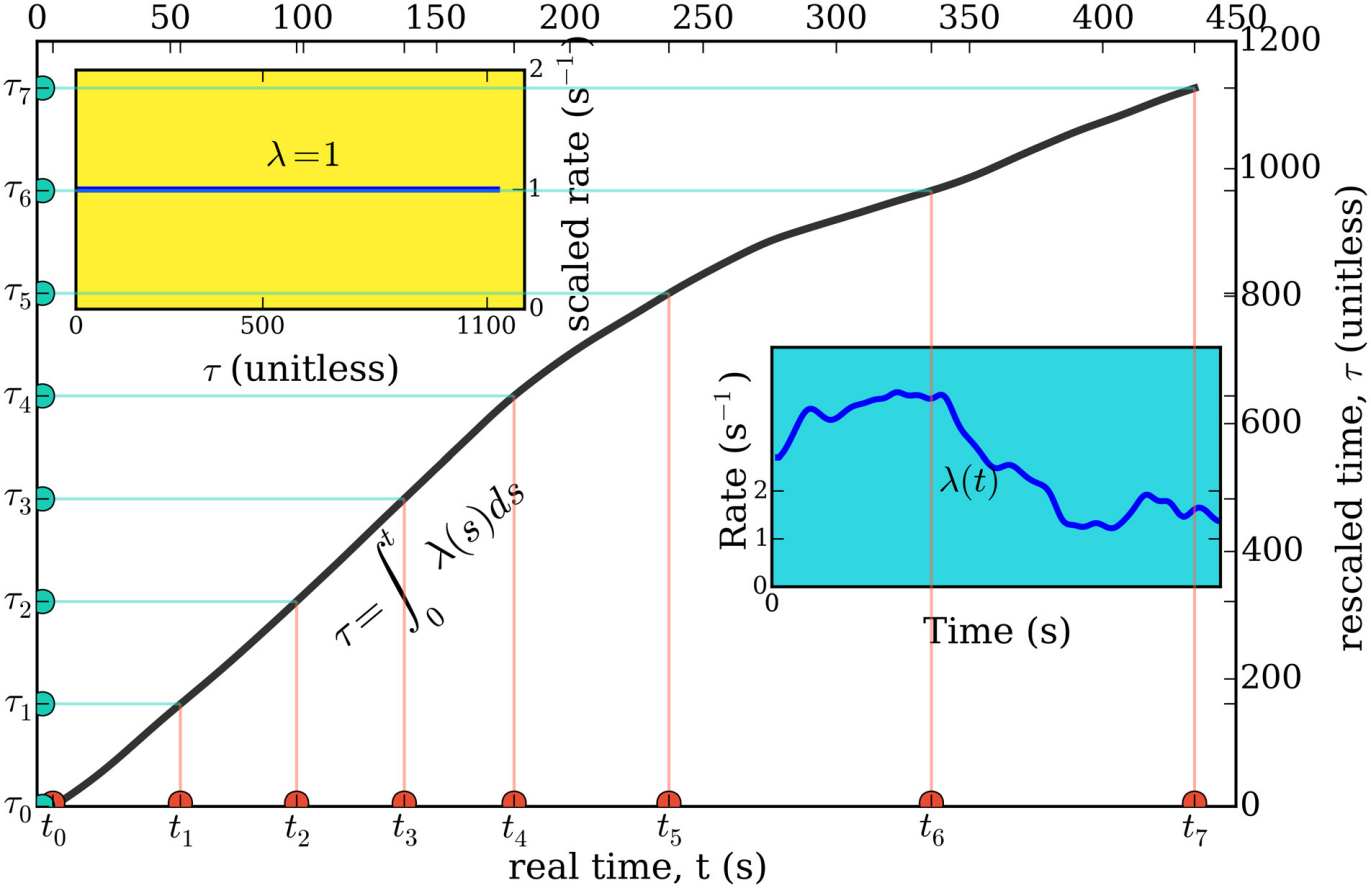
Representation of the time rescaling theorem [64, 65] applied to our problem. The right inset plot shows the rate of one of the studied chromaffin cells. The real time *t* (*x*-axis) is transformed into a rescaled time *τ* (*y*-axis), as a function of which the spike series has a constant rate equal to 1. The solid curve shows the transformation from *t* to *τ*.

Intuitively, this transformation stretches time in proportion to the spike rate λ(*t*), so that when the rate λ(*t*) is high, spike intervals are lengthened and when λ(*t*) is low, spike intervals are compressed. (See Fig 2). Thus, the time rescaling transformation always maps to spike sequences of constant rates. To get back to the original rate function of the cell, one only needs to multiply the rescaled time *τ* by the estimated rate function of the active cell.

## 4 Statistical analysis of vesicle-release events

The stochastic dynamics of the sequence of releases can be described by computing the corresponding probability distribution [66]. For this purpose we first estimated the distribution of spikes by performing a statistical analysis, and then we explored the suitable mathematical models to represent that distribution. Since a spike sequence is a point process, it can be uniquely characterized either by the interspike times or by the spike counts, as the corresponding variables related to each other. We analyzed both variables in order to efficiently look for a suitable point-process model. The analysis we performed are based on exploratory data analysis technique [67].

### 4.1 Interspike-time histograms

For a spike sequence {*t*_0_, *t*_1_, …*T*} on the interval (0, *T*], an interspike time is defined as the time difference between subsequent spikes (*δt*_*i*_ = *t*_*i* + 1_ − *t*_*i*_). The interspike time is a continuous variable with a sequence of realizations, {*δt*_0_, …, *δt*_*N* − 1_}. We constructed the interspike time sequences and calculated the summary statistics as mean, median, variance, standard deviation and coefficient of variation. As a first estimate of the time-interval probability distribution we computed the interspike time histograms. Histograms allow one to obtain information on the underlying distribution pattern, including the center location, the spread, the outliers, the skewness, and the presence of multiple modes in the data. This preliminary knowledge of summary statistics and data distribution pattern helps in the selection of appropriate probabilistic models. For a sufficiently large data set, a histogram provides a good estimate of the probability distribution. To construct a histogram, we separated the interspike time data into N number of bins of equal bin-widths and plotted the number of observations in each bin. Since the shape of a histogram is sensitive to a bin-width, for any sequence with the mean *μ*, the standard deviation *σ* and *N* < 1000, we computed the bin-width *h* as

$$\begin{array}{c} h=\left(\mu +2.85\sigma \right)/N^{1/2}. \end{array}$$ (4)

For large sequences we used a bin-width from the combination of the above expression and the scaled Scott’s rule [68] as,

$$\begin{array}{c} h=\left(\mu +2.85\sigma \right)\left(\frac{1}{2N^{1/2}}+\frac{1}{2N^{1/3}}\right). \end{array}$$ (5)

**Fig 3:**
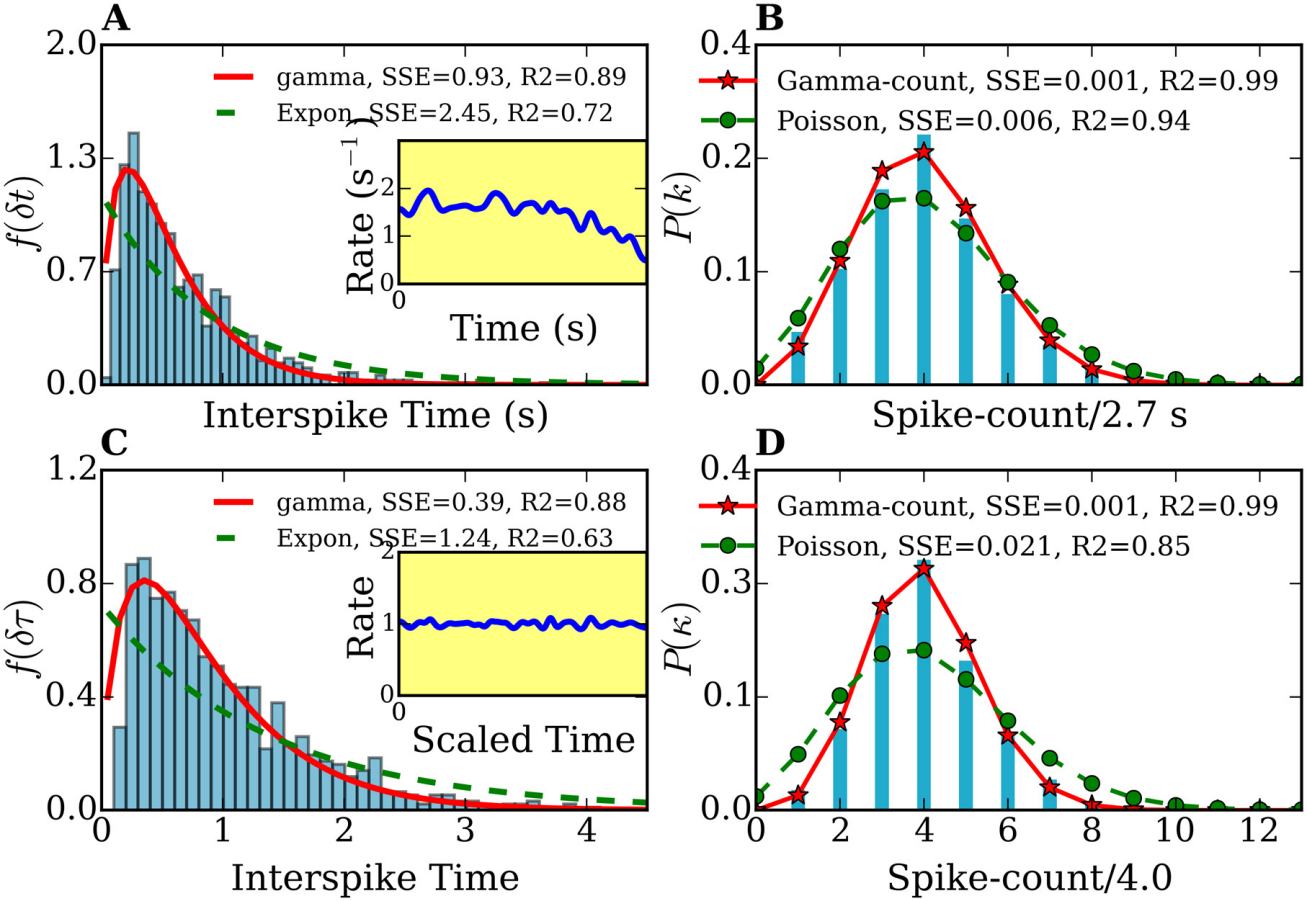
Interspike time- and frequency-count histograms of a single-cell record. (A), (C) Interspike time histograms *f*(*δt*) and *f*(*δτ*) before and after time rescaling, respectively. (B),(D) Frequency-count histograms *P*(*k*) (for a time-interval of *T* = 2.7 s width) and *P*(*κ*) (for a rescaled time-interval of $\bar{\tau }=4.0$ width) before and after time rescaling, respectively. The histograms *f*(*δt*) and *f*(*δτ*) are fitted with gamma (solid lines) and exponential distributions (dashed lines). The frequency-count histograms *P*(*k*) and *P*(*κ*) are fitted with the gamma-count (see text, solid lines) and Poisson distributions (dashed lines). The number of spikes was 914. Inset plots in (A) and (C) show the spike rates of the original and time-rescaled spike series, respectively.

In Fig 3(A) we show an example of a histogram for the distribution of spike intervals taken from our amperometric measurement.

### 4.2 Frequency-count histograms

For a spike sequence {*t*_0_, *t*_1_, …*T*} on the interval (0, *T*], the spike count in an interval *ΔT* is the number of spikes occurring in the interval [*t*, *t* + *ΔT*). To calculate the count data, we performed a partition of the spike sequence into M bins, with a bin-width, *ΔT*, and counted the number of the spikes occurring within each bin. This resulted in a list of integer count numbers {*n*_1_, *n*_2_, …, *n*_*M*_}. We performed the quantitative analysis by calculating the summary statistics similar to the case of the interspike times. Then we constructed a frequency plot, which is more suitable for analyzing a discrete count variable using graphical analysis. In our long amperometric recording data, the frequency plots provide good estimates of the probability distributions of the spike counts. To visualize the spike-count histogram we simply plotted the frequency of each count against its value (see, for example, Fig 3(B)). We chose the bin-width *ΔT* = 4*μ*, where *μ* is the mean of interspike time of the sequence.

### 4.3 Probabilistic models

A proper probabilistic model must be able to describe the release sequence and also to connect it to a microscopic picture of the process. In this work, we chose the ‘stationary stochastic’ point process, since each spike sequence obtained from an amperometric recording is a point process datum by definition. A point process can be represented by a sequence, {*t*_*i*_}, on the interval, (0, *T*], with the ordering *t*_0_ < *t*_1_ < … < *t*_*n*_ < *T*. It is characterized by an intensity function (rate function) λ(*t*). Stationary point processes are mathematically well defined in probability theory. There exist analytic expressions for the probability distribution functions and moments (e.g. mean, variance, etc). A stationary model can be easily extended to an arbitrary non-stationary model described by a time-dependent rate function λ(*t*).

#### Stationary Poisson process

Stationary (or homogeneous) Poisson processes are the most simple point processes. They characterize purely random processes with constant rate parameters λ. An example of Poisson process is radioactive decay, where naturally occurring gamma rays detected in a scintillation detector are randomly distributed in time. If vesicle releases in Chromaffin cells are Possion processes, then the probability of a release event at any given time should be independent of all other events. Then the interspike times, *δt*, between neighboring spikes, (*t*_*i*_, *t*_*i*_ + *δt*), would be homogeneously distributed, and their probability distribution function would be given by the exponential form

$$\begin{array}{c} f\left(\delta t\right)=\lambda e^{-\lambda \delta t}. \end{array}$$ (6)

Accordingly, the probability distribution of the number of spikes *k* per fixed time interval *T* would follow Poisson statistics,

$$\begin{array}{c} P\left(k\right)=\frac{{\left(\lambda T\right)}^{k}}{k!}e^{-\lambda T}. \end{array}$$ (7)

A Poisson distribution fulfills the equation

$$\begin{array}{c} \mu =\sigma^{2}=\lambda , \end{array}$$ (8)

where *μ* and *σ* ^2^ are the mean and the variance, respectively. This follows from the unit Fano factor which characterizes the variability in the spike count, $\frac{\sigma^{2}}{\mu }=1$.

Analogously if the time-rescaled spike sequence would follow a stationary Poisson process, its interspike time distribution and spike count distribution are.

$$\begin{array}{ccc} f(\delta \tau ) & = & \lambda e^{-\lambda^{\prime }\delta \tau },\\ P(\kappa ) & = & \frac{{\left(\lambda^{\prime }\bar{\tau }\right)}^{\kappa }}{\kappa !}e^{-\lambda^{\prime }\bar{\tau }}, \end{array}$$ (9)

where $\bar{\tau }$, *κ* and λ′ are the interval, the number of spikes within $\bar{\tau }$ and the constant spike rate of the time-rescaled spike sequence.

Poisson processes describe for purely non-deterministic stochastic systems. If the neurotransmitter release process is not purely random, but partly-deterministic, then other history dependent point processes, e.g. a renewal point process, would better characterize its spike sequences.

#### Gamma process

A more appropriate point process to describe our sequences is the gamma process. In a gamma process, independent and identically distributed interspike times exhibit more (history dependent) order than a Poisson process. Its probability distribution function follows a two-parameter gamma distribution, which is given by

$$\begin{array}{c} f\left(\delta t,p,\theta \right)=\frac{{\left(\delta t\right)}^{p-1}e^{-\frac{\delta t}{\theta }}}{\theta^{p}\Gamma \left(p\right)}\;\;\text{for}\;\delta t>0\;\text{and}\;p,\theta >0. \end{array}$$ (10)

where *p* and *θ* are the scale and shape parameters. Γ(*p*) is the gamma function evaluated at *p*. Note that if *δt* ∼ Gamma(1, λ), then *δt* has an exponential distribution with rate parameter λ. It is important to point out that the gamma distribution reflects a certain correlation between events, in contrast to the exponential distribution.

Accordingly, the probability distribution of the number of spikes *k* per fixed time interval *T* follows the gamma-count distribution. The gamma-count distribution for *P*(*k*) is derived from the gamma distribution for *f*(*δt*) in the same way as a Poisson distribution is derived from the exponential one. The gamma-count distribution is given by [69])

$$\begin{array}{c} P(k)=G(pk,\beta T)-G(pk+p,\beta T)\;k=0,1,2,\ldots , \end{array}$$

where *G*(*pk*, *βT*) is the incomplete gamma function defined as,

$$\begin{array}{c} G\left(pk,\beta T\right)=\frac{1}{\Gamma (pk)}\int_{0}^{\beta T}u^{pk-1}e^{-u}du \end{array}$$ (11)

*β* = 1/*θ*, and *p* are the shape and scaled parameters receptively.

Analogously for the time-rescaled spike sequence, the interspike time distribution and the spike count distribution have the forms of gamma distribution *f*(*δτ*) and gamma-count distribution *P*(*κ*) respectively. where the time $\bar{\tau }$, the number of spikes *P*(*κ*) within $\bar{\tau }$ and the constant spike rate λ′ are the time-rescaled quantities.

## 5 Results

In the previous sections, we described how to map our amperometric traces into spike sequences of exocytosis events and how to perform a statistical analysis. In this section, we present and discuss the results. The analysis was performed on 29 of long amperometric recordings with clear signals.

### 5.1 Exocytosis rate

As mentioned in section 3.2, the calculated rate function of each experiments varies in its duration and magnitude (with mean frequencies ranging from 0.6–2.5 Hz), but share a similar feature of a temporary plateau followed by an S-shape decay (see example in Fig 1). We fitted each rate with a logistic function, $r(t)=\frac{r_{0}}{1+e^{-\beta (t-\mu )}}+r_{f}$. *r*_0_, *r*_*f*_, *β* and *μ* are the initial rate, the final rate, the rate-decay constant, and the time of midpoint where the rate reaches its mean value respectively. Notice that the logistic model works well in describing a population decay in a system of diffusing particles confined in a cage with absorbing boundary [70, 71]. Thus, the fact that exocytosis in the sustained regime exhibits logistic-decay rates indicates a correlation between the decreasing of the population of vesicles and neurotransmitter releases. If the exocytosis-rate would be governed by other mechanisms (like priming and fusion), it would not be well described by a logistic population decay model.

### 5.2 Statistical analysis of single measurements

Using the methods described in section 4 we determined the probability distributions of individually spike sequences and of their stationary counterparts, the time-rescaled spike sequences. We calculated the summary statistics, the interspike time histograms and the frequency-count plots. Fig 3(A) and 3(C) show the distribution *f*(*δt*) for a spike sequence directly obtained from one of our amperometric measurements and *f*(*δτ*) from the rescaled spike sequence, respectively. The original and rescaled release rates are shown in the inset plot of 3 (A). Note the decaying behavior of the rate in the original spike sequence, characteristic for *ex-vivo* experiments. In contrast, the rate of the rescaled spike sequence remains around 1 with small fluctuations. These fluctuations are due to the finite size of the peak-broadening used for the time-rescaling transformation. Fig 3(B) and 3(D) show the frequency-count histograms for the original (*P*(*k*)) and time rescaled (*P*(*κ*)) spike series, i.e., the probability distribution for the occurrence of *k*(*κ*) spikes within a fixed time interval *T*($\bar{\tau }$) before and after time rescaling, respectively. Histograms corresponding to the other 28 measurements show similar features.

In order to characterize the release process on the basis of the statistics of the spike sequences it is first necessary to find the probabilistic model which best reproduce the histograms of Fig 3 and those corresponding to the other amperometric measurements we performed. Note that if, for instance, the sequence of neurotransmitter releases would be purely random, the number of exocytosis events per fixed time interval (frequency-count histogram) would follow a Poisson distribution. Therefore, and in order to determine the degree of ‘randomness’ in the analyzed spike series, we analyzed first our histograms in the framework of Poisson statistics. For this purpose, we fitted the interspike time histograms with exponential distributions and the spike-count histograms with Poisson distributions. We found that the statistics of all measurements and their rescaled spike sequences deviates considerably from that of Poisson processes (e.g. see Fig 3). From Fig 3(A) it is clear that the interspike time distribution *f*(*δt*) cannot be fitted with an exponential function [Eq (6)]. Moreover, this deviation cannot be attributed to the fact that the rate decreases, since the interspike time histogram *f*(*δτ*) of the time-rescaled sequence also deviates from an exponential behavior (see Fig 3(C)). The discrepancies with the Poisson model are confirmed in the frequency-count histograms of Fig 3(B) and 3(D). Similar discrepancies were found by analyzing the histograms corresponding to the other 28 amperometric measurements performed. All fits yielded an index of dispersion smaller than one (*σ* ^2^/*μ* < 1). The underdispersion reflects some regularity in the spike pattern, indicating that the releases processes are not purely random. Thus, **Poisson process are not suitable to model our data**.

To take into account the partly deterministic behavior of release sequences, we considered gamma and log-normal processes as possible models to fit our data. Both are two-parameter point processes which allow underdispersion. We combined the analysis of the interspike-time and the frequency-count variables by minimizing the sum of the squared errors (SSE) [72]. We fitted both variables’ histograms to describe the same point process. We found that the interspike-time histograms of the original and time-rescaled spike sequences are very well described by a gamma distribution as illustrated in Fig 3(A) and 3(C). We repeated the same fitting procedure for the other 28 spike sequences and confirmed that fact. A measure of the quality of the fit is given by the coefficient of determination *R* ^2^[72]. The average value of *R* ^2^ over the 29 measurements was <*R* ^2^ > = 0.89 for the original spike series and <*R* ^2^ > = 0.93 for the time-rescaled sequences (<*R* ^2^ > = 1 would correspond to a perfect fit).

The histogram of Fig 3(A) exhibits an interspike-interval mean *μ* = 0.68 s and an interspike-interval standard deviation *σ* = 0.56 s. The parameters used for the fits shown in Fig 3(A) are: (i) exponential fit: λ = 1.14 s^−1^, (ii) gamma fit: *p* = 1.58 and *θ* = 0.36 s. The histogram of Fig 3(C) shows an interspike-interval mean *μ*′ = 1.00 and an interspike-interval standard deviation *σ*′ = 0.72. In Fig 3(C) the parameter for the exponential fit is λ′ = 0.73 and the parameters for the gamma fit are *p*′ = 1.65 and *θ*′ = 0.54. We stress here that only the spike sequence with constant rates (e.g. a time-rescaled sequence) could be modeled with a stationary point process. However, for this specific measurement, the rate function does not change much and its influence on the distributions is not large. This can be observed by comparing Fig 3(B) with Fig 3(D).

### 5.3 Spike statistics of an ensemble of cells

**Fig 4:**
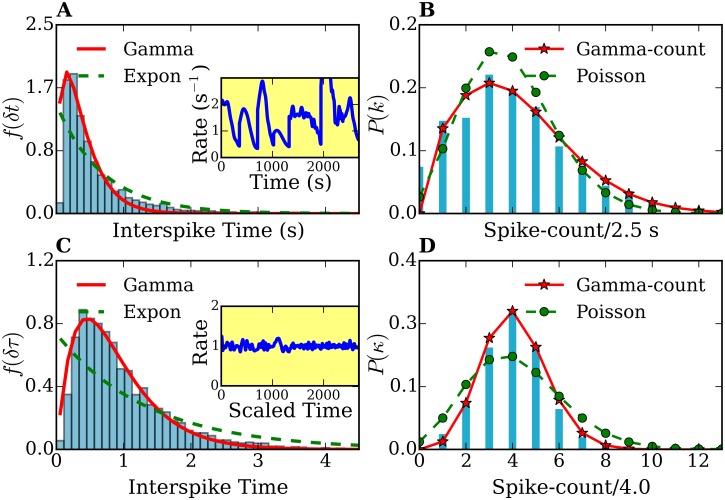
Interspike-times histograms of a combined data set of 7 different cells. *f*_7_(*δt*) (A) and *f*_7_(*δτ*) (C), and frequency-count histograms *P*_7_(*k*) (for a time interval *T* = 2.5 s) (B) and *P*_7_(*κ*) (for a rescaled time interval $\bar{\tau }=4.0$ (D)) before and after time-rescaling. The histograms *f*_7_(*δt*) and *f*_7_(*δτ*) are fitted with gamma (solid lines) and exponential distributions (dashed lines). The frequency-count histograms *P*_7_(*k*) and *P*_7_(*κ*) are fitted with the gamma-count (see text, solid lines) and Poisson distributions (dashed lines). Inset plots in (A) and (C) show the spike rates of the original and time rescaled spike series, respectively. The number of spikes used was 4252.

Fig 4 shows the probability distributions *f*_7_(*δt*) (*f*_7_(*δτ*)) and *P*_7_(*k*) (*P*_7_(*κ*)) for a combined dataset consisting of seven consecutively placed independent spike series obtained from seven different cells. Defining $T_{i}^{M}$ the measurement time for the *i* ^th^ cell, the total rate of the ensemble is constructed as λ(*t*) = λ_*i*_(*t*) for $T_{i-1}^{M}<t\leq T_{i}^{M},$ *i* = 1, …7 ($T_{0}^{M}=0$). Thus, the total time considered in Fig 4 is $T_{T}^{M}=\sum_{1}^{7}T_{i}^{M}$. Note that before time rescaling [Fig 4(A) and 4(B)] the rate λ(*t*) (see inset plots) is strongly fluctuating. After rescaling, the rate stays around 1. As a consequence, histograms after time rescaling [Fig 4(C) and 4(D)] show much less statistical fluctuations. Remarkably, the combined histograms of 7 cells *f*_7_(*δt*) (before rescaling) and *f*_7_(*δτ*) (after time rescaling) are also well fitted by a gamma distribution, while *P*_7_(*k*) and *P*_7_(*κ*) are well described by a gamma-count distribution, and differ from exponential and Poisson distributions. In particular, the gamma and gamma-count distribution seem to describe *f*_7_(*δτ*) and *P*_7_(*κ*), respectively, with a high degree of agreement.

The histogram of Fig 4(A) exhibits mean *μ* = 0.63 s and standard deviation *σ* = 0.77 s, The parameters used for the fits shown in Fig 4(A) are: (i) exponential fit λ = 1.43 s^−1^, (ii) gamma fit: *p* = 1.61 and *θ* = 0.24 s. In Fig 4(C) the mean is *μ*′ = 1.00, and standard deviation *σ*′ = 0.74. The parameter for the exponential fit is λ′ = 0.74, whereas for the gamma fit we obtained *p* = 2.10 and *θ*′ = 0.43. The fitting of histograms in Figs 3 and 4 were performed by a nonlinear least-squares optimization using the scipy library [73].

The fact that all studied cells and their ensemble show similar interspike interval and spike-count statistics differing from exponential (Poisson) behavior definitively reveals that releases of vesicles coming from the reserve pool must play an important role in our measurements. Moreover, their interspike-time and count distributions are well described by underdispersed gamma processes, indicating regularity in the release patterns. This implies partly deterministic behavior of the release processes.

### 5.4 Connection between amperometry and vesicle transport

When exocytosis starts as a response to a stimulus, the pools of already docked and primed vesicles are fully or partially depleted, or at least considerably reduced within 1–10 s [4]. This means that although vesicles of the reserve pool are stimulated after that time, releases from the pre-docked and primed vesicles might also be present in the measured signals. Therefore, the most general assumption is that the amperometric signal contains a superposition of releases coming from both the reserve pool (i.e., from those vesicles we are interested in) and from the pool of initially docked/primed vesicles. Although Hugo et al. [52], reported that around 15% of the vesicles at the membrane do not release (dead-end vesicles), the influence of prime/fusion reaction times on the amperometric signals cannot be *a priori* neglected. Therefore, a model considering both transport and fusion times is needed to interpret the spike series obtained from experiment. In the next section we present such a model.

## 6 Langevin Simulations

Based on the statistical analysis of our experimental results we propose a model for exocytosis of catecholamines in chromaffin cells using Langevin simulations.

### 6.1 Stochastic model of vesicle transport

We propose the following biophysical picture, which relies on our results and analysis of the previous sections: catecholamines release events in the sustained regime can be viewed as successive arrivals of vesicles at the membrane, which then undergo fusion and lead to exocytosis. Then we can assign a spike to each arrival and in this way mimic the amperometric peaks. Assuming similar processes for all release events, the times of successful arrivals of the vesicles at membrane result in a spike sequence which corresponds to the time rescaled spike sequences derived from our experimental signals. Corrections due to prime/fusion reaction times will be included later (see Section 6.4).

In order to simulate the arrival of vesicles at the membrane, one can describe vesicle motion in the liquid cellular medium by Langevin equations of the form

$$\begin{array}{c} m_{i}\frac{d}{dt}v_{i}\left(t\right)=-\frac{\partial }{\partial r_{i}}W\left(r_{i}\right)-\gamma v_{i}\left(t\right)+\sigma \xi \left(t\right), \end{array}$$ (12)

where **v**_*i*_ is the velocity of *i* ^*th*^ vesicle and *γ* is the friction coefficient. *W*(**r**) is a potential to which vesicles are subject to. *ξ*(*t*) is a stochastic microscopic force produced by the environment, with a coupling coefficient given by [66]

$$\begin{array}{c} \sigma =\sqrt{2k_{B}T\gamma } \end{array}$$ (13)

for Gaussian white noise, where *k*_*B*_ is the Boltzmann constant and *T* the absolute temperature. The friction coefficient can be related to the diffusion coefficient by

$$\begin{array}{c} D=\frac{2k_{B}T}{\gamma }. \end{array}$$ (14)

**Fig 5:**
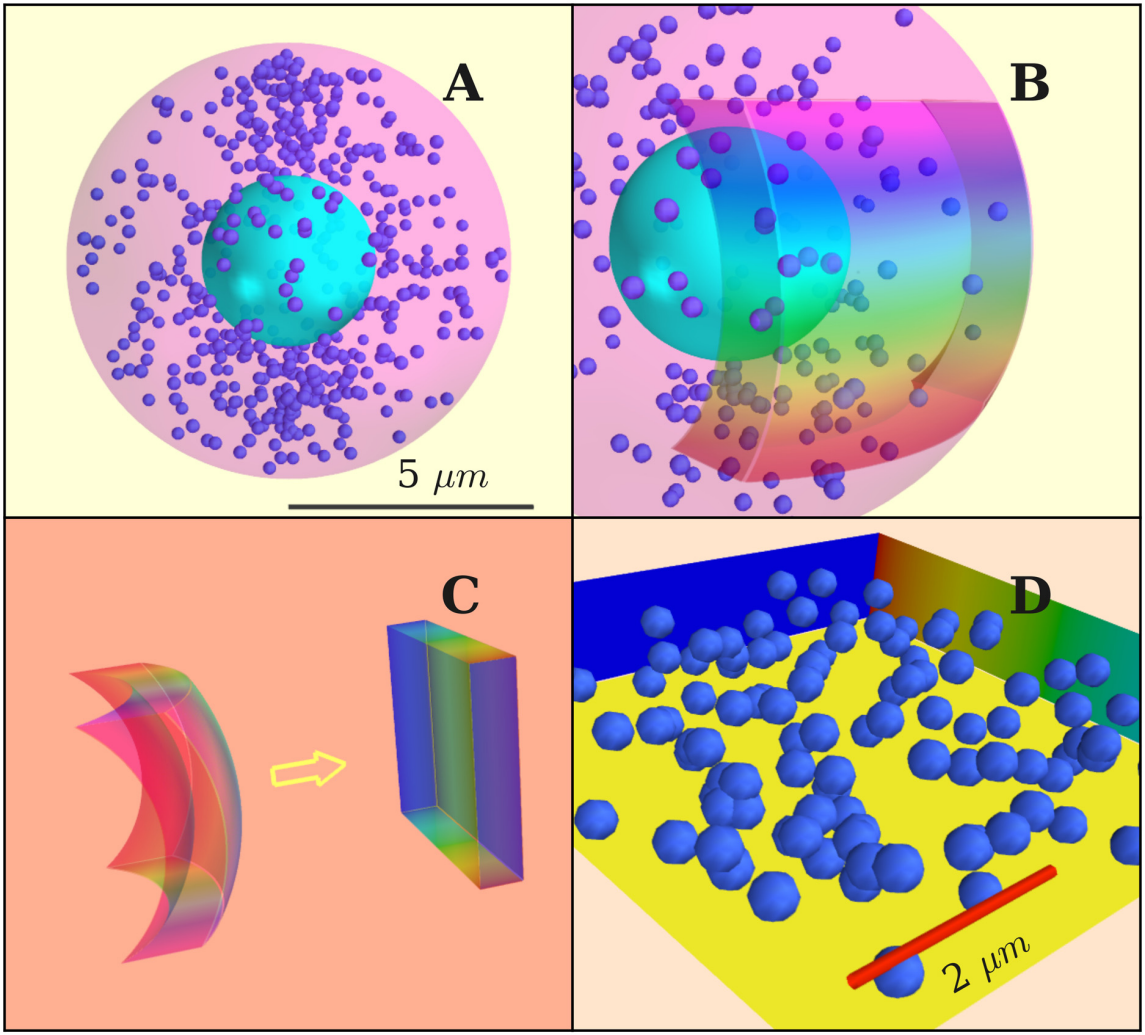
(A) Simulated cell with diameter 10 *μ*m. The diameter of vesicles is 300 nm. (B) Model for active zone, chosen as a thin layer close to cell membrane. (C) Map of the active zone onto a cuboid. (D) Cell volume in the simulations. Scale bar: 2 *μ*m. Vesicles elastically bounce from all walls except from the membrane, where fusion is assumed to occur.

Thus, we model vesicles as diffusive particles in a bounded domain. Whenever a vesicle reaches the membrane, a release event is assumed. Fig 5(A) sketches our model for a chromaffin cell. In Fig 5(B) we represent the portion of the cell which can be accessed by the electrode in an experimental setup. Only exocytosis events in this region can be captured in an amperometric recording. For simplicity, we assume that this region has a cuboidal shape [Fig 5(C)], which is topologically equivalent to the curved layer of Fig 5(B). The membrane is thus represented by a square [Fig 5(D)].

Since the cytosol is highly viscous, we can assume an overdamped regime $m_{i}\frac{d}{dt}{\text{v}}_{i}(t)=0$, and Eq (12) becomes

$$\begin{array}{c} v_{i}\left(t\right)=\frac{F(r_{i})}{\gamma }+\frac{\sigma }{\gamma }\xi \left(t\right), \end{array}$$ (15)

where *F*(**r**) = −∂*W*(**r**)/∂**r**_*i*_.

We performed two kinds of simulations. First, we assumed no external potential, i.e., pure diffusive (Brownian) motion in order to prove that purely random motion of vesicles does result in Poisson processes. In a second step, we included an attractive potential in the direction of the membrane as deterministic component.

### 6.2 Vesicles as free Brownian particles

We considered the case of free Brownian particles with no external potential, i.e., *W*(**r**) = 0. For the diffusion coefficient of vesicles in the cytoplasm we used *D* = 3.22 × 10^−14^ m^2^ s^−1^[30, 32]. We determined the number of vesicles within our simulation volume as follows.

Considering the cell as a sphere of radius *R* = 5 *μm* [Fig 5(A)], taking into account that electron microscopy and reflection fluorescence microscopy measurements yielded a vesicle density beneath the membrane’s surface of ∼.45 − 1.7 vesicles *μ*m^2^, and that the active zone has a depth of 120 − 300 nm, we estimated a vesicle density of 2.09 vesicles/*μ*m^3^[23, 28, 30, 74]. Vesicles were considered as hard spheres of radius equal to 150 nm. Other cell elements were not physically present in our simulation volume, but they were effectively included through the noise term of the Langevin equations. According to Refs. [33, 36, 75], the main processes involved in exocytosis occur at the cell’s boundary. We assumed this region to coincide with our simulation volume [Fig 5(C)]. The faces of the cuboid are treated as hard walls except the one describing the membrane. The simulations were conducted as follows. First, we distributed the vesicles randomly inside the cuboid. Then we let them evolve by integrating Eq (12). All collisions were treated as elastic with the exception of collisions of vesicles with the membrane. The hits of the vesicles with the membrane were recorded as exocytosis events. The number of vesicles in the volume was kept constant during the simulations. After a hit with the membrane, the vesicle involved was taken out and replaced by another vesicle entering the cuboid at a randomly chosen position. Notice that Langevin simulations describe stationary processes and therefore the histograms *t*(*δt*) and *P*(*k*) obtained from them can be directly compared to the time-rescaled spike sequences obtained from experiment. However, for the sake of comparison and in order to normalize the (constant) rate to 1, we also performed time-rescaling on the sequences obtained from our simulations.

In Table 1 we give an overview of the parameters used in our simulations. Since our amperometric experiments were conducted at room temperature(see section 2.2), we set *T* = 296 K in all simulations.

**Table 1:** Parameters used in our Langevin simulations.

Parameter		Value
Diffusion coefficient	D	3.22 × 10^−14^ m^2^ s^−1^[30, 32]
Temperature	*T*	296 K
Vesicle’s radius	r	150 nm [23]
Density	*ρ*	2.09 vesicles/*μ*m^3^[30, 74]
Volume	L	*L*_*x*_ = *L*_*z*_ = 4.4 *μ*m, *L*_*y*_ = 1 *μ*m

**Fig 6:**
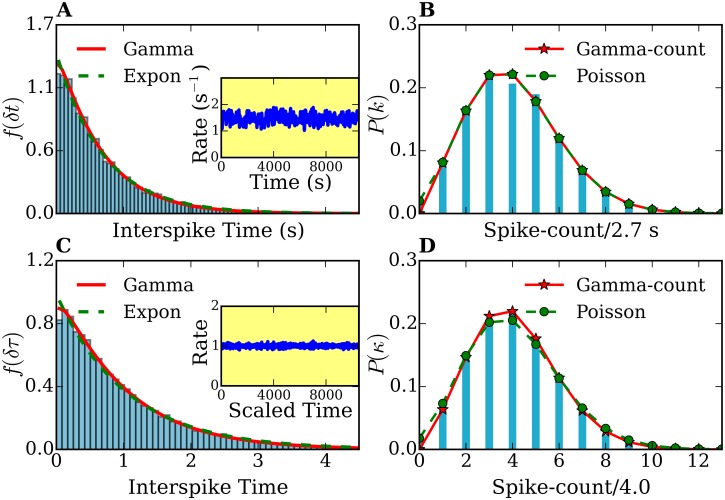
Interspike-time and frequency-count histograms of a simulation of free Brownian vesicles. (A), (C) Interspike-time histograms *f*(*δt*) and *f*(*δτ*) before and after time rescaling, respectively. (B), (D) frequency-count histograms *P*(*k*) (for a time interval of *T* = 2.7 s width) and *P*(*κ*) (for a rescaled time interval of $\bar{\tau }=4.0$ width) before and after time rescaling, respectively. The histograms *f*(*δt*) and *f*(*δτ*) are fitted with gamma (solid lines) and exponential distributions (dashed lines). The frequency-count histograms *P*(*k*) and *P*(*κ*) are fitted with the gamma-count- (see text, solid lines) and Poisson distributions (dashed lines). The number of spikes was 15504. Inset plots in (A) and (C) show the spike rates of the original and time rescaled spike series, respectively.

The results of the simulations in the purely Brownian regime are shown in Fig 6. The inset figures in Fig 6(A) and 6(C) show the exocytosis rate in the original and time rescaled spike sequences obtained from the simulations. Clearly, the only effect of time rescaling is the normalization of the rate to 1. There is no influence of the time-rescaling transformation on the histograms. The fact that *f*(*δt*) and *P*(*k*) can be perfectly described by an exponential and by a Poisson distribution, respectively, confirms our idea that pure Brownian motion of vesicles coming from the reserve pool results in release-event statistics governed by Poisson processes. The histogram of Fig 6(A) is characterized by *μ* = 0.68 s, *σ* = 0.67 s, The parameters used for the fits are: (i) λ = 1.44 s^−1^, *p* = 1.08 and *θ* = 0.61 s. Fig 6(C) are characterized by *μ*′ = 1.00 and *σ*′ = 0.96. The parameters used for the fits are λ′ = 0.96, *p*′ = 1.10 and *θ*′ = 0.90.

### 6.3 Diffusive vesicles under an attractive potential towards the membrane

The simulation set up was similar to the free Brownian case, except that an additional attractive potential toward the membrane was assumed. We used an harmonic potential leading to the following forces:

$$\begin{array}{ccc} F_{x} & = & F_{z}=0\\ F_{y} & = & \alpha (y-y_{0}), \end{array}$$ (16)

where *y* is the direction perpendicular to the membrane, which is placed at *y*_0_.

**Fig 7:**
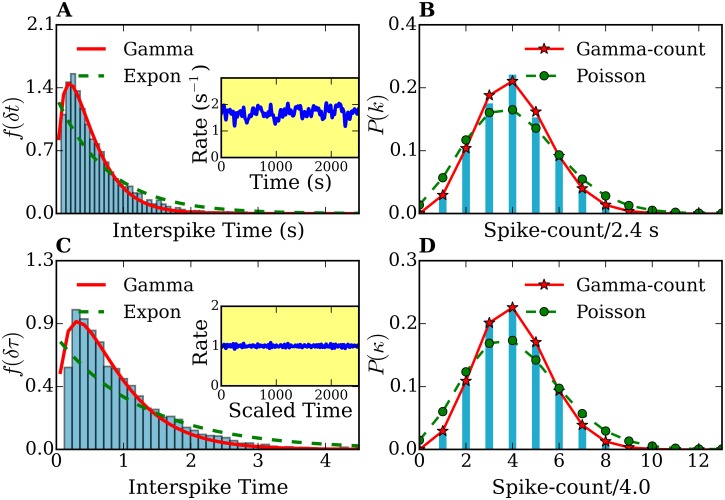
Interspike-time and frequency-count histograms of a simulation of diffusive vesicles under an attractive harmonic potential, before and after time-rescaling. (A), (C) Interspike-times histograms *f*(*δt*) and *f*(*δτ*) before and after time rescaling, respectively. (B),(D) frequency-count histograms *P*(*k*) (for a time interval of *T* = 2.3 s width) and *P*(*κ*) (for a rescaled time interval of $\bar{\tau }=4.0$ width) before and after time rescaling, respectively. The histograms *f*(*δt*) and *f*(*δτ*) are fitted with gamma-(solid lines) and exponential distributions (dashed lines). The frequency-count histograms *P*(*k*) and *P*(*κ*) are fitted with the gamma-count (see text, solid lines) and Poisson distributions (dashed lines). The number of spikes was 4237. Inset plots in (A) and (C) show the spike rates of the original and time rescaled spike series, respectively.

The value of the parameter *α* which best reproduces experiment was 1.275 × 10^−13^ N/m. Results of the simulations are shown in Fig 7. For the sake of comparison and in order to normalize the rate, we also performed here time-rescaling.

The histogram of Fig 7(A) is characterized by *μ* = 0.59 s and *σ* = 0.48 s, The parameters used for the fits are λ = 1.31 s^−1^, *p* = 1.63 and *θ* = 0.30 s. The histogram of Fig 7(C) is characterized by *μ*′ = 1.00 and *σ*′ = 0.78. In Fig 7(C) the parameters for the fits are λ′ = 0.76, *p*′ = 1.59 and *θ*′ = 0.52.

Remarkably, the interspike-time and frequency-count histograms obtained from our simulations with an attractive force derived from an harmonic potential can be better fitted by gamma than by Poisson processes. By comparing this fact with our statistical analysis of the experimental results we conclude that our simulations with an attractive harmonic potential can qualitatively reproduce the experimental trend. From our simulations and experiments one can clearly see a direct connection between vesicle transport and the amperometric spike series. Below, we investigate the influence of priming/fusion reaction times.

### 6.4 Simulations including an attractive potential and priming/fusion statistics

**Fig 8:**
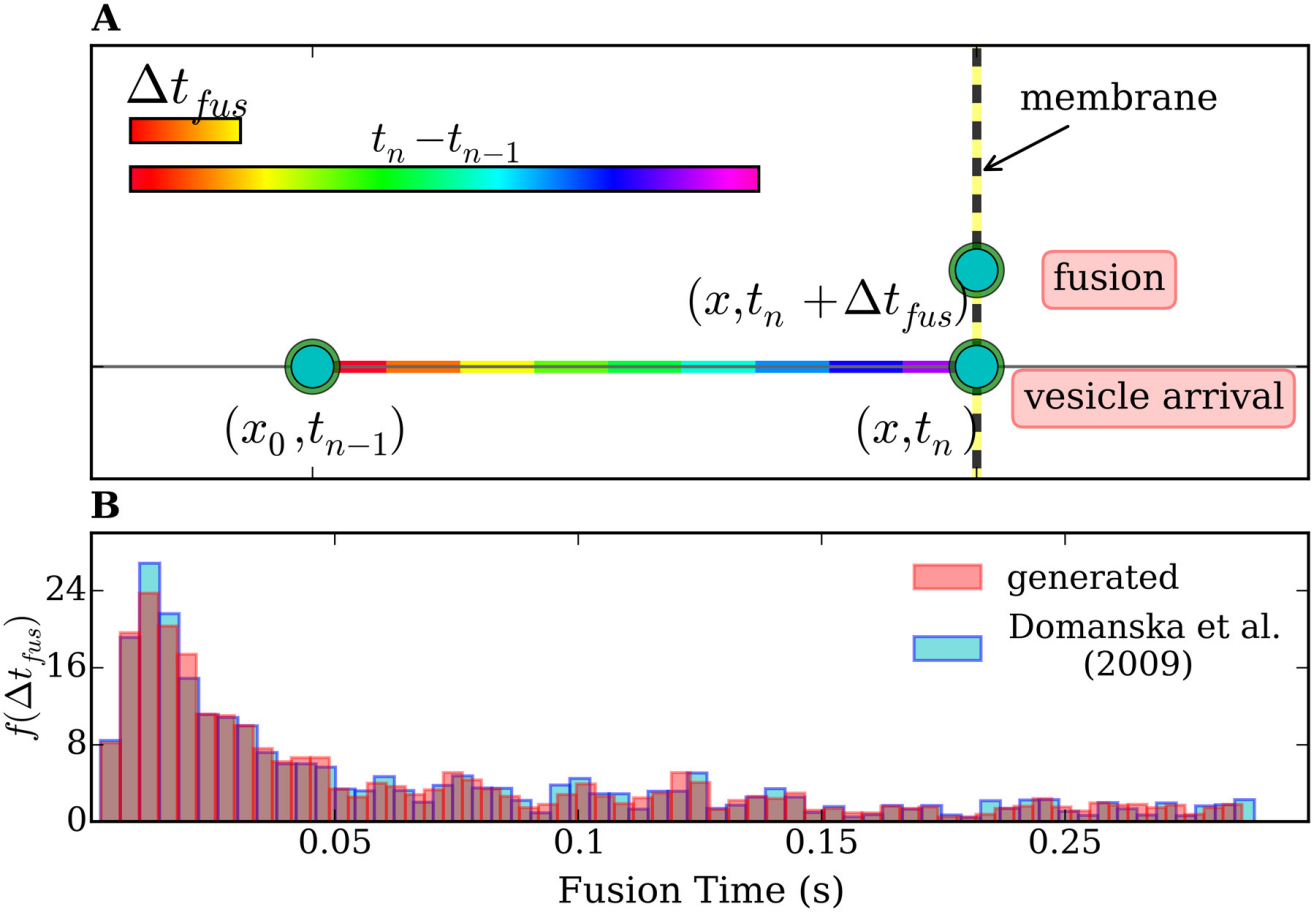
(A) Projection of the trajectory of the vesicle responsible for the *n* ^*th*^ event on the axis perpendicular to the membrane. At the time *t*_*n* − 1_, which refers to the (*n* − 1)^*th*^ event, the vesicle is at a distance *x*_0_ from the membrane. The vesicle arrives at the membrane at time *t*_*n*_ and releases at time *t*_*n*_ + *Δt*_*fus*_. A difference in the time scale of the priming/fusion reaction and the transport process is shown in the color bars. (B) Normalized frequency histogram *f*(*Δt*_*fus*_) (number of fusion events vs *Δt*_*fus*_) taken from Ref. [35] (blue) and the normalized frequency histogram of our generated *Δt*_*fus*_ (pink), for comparison. The time scale for fusion *Δt*_*fusion*_ (see x-axis) is at least one order of magnitude smaller than *t*_*n*_ − *t*_*n* − 1_ (see previous figures). The difference in the time scales is schematically represented by the color bars in A.

In order to include the priming/fusion time statistics in our numerical model we proceed as follows. We define a ‘fusion time’ defined as a lag time *Δt*_*fus*_ between docking and fusion. The vesicles arriving at the membrane have to wait a time *Δt*_*fus*_ before generating an event which counts for the spike series. To the best of our knowledge, no direct measurement of fusion times in chromaffin cells was performed so far. For SNARE-mediated vesicle fusion, however, Domanska et al. observed the lag times between docking and fusion (*Δt*_*fus*_) and characterized it by a probability histogram *f*(*Δt*_*fus*_), obtained as the number of fusion events as a function of the lag times [35]. To include this lag times in Langevin simulations one needs a variable *Δt*_*fus*_ (Fig 8(A)) fulfilling the statistics found by Domanska et al. in Ref. [35]. Therefore, we first constructed a continuous probability density function out of the histogram of Ref. [35] by convolving it with a Gaussian window and obtained the cumulative distribution. The standard deviation for the Gaussian kernel was 0.2 of the bin-width of the histogram. Then, we applied the inverse-transform method for generating random variables [76] to get *Δt*_*fus*_. The distribution of our obtained random-generated fusion times is shown in Fig 8(B). It reproduces the histogram from Ref. [35]. In this way we were able to introduce the additional variable *Δt*_*fus*_ and study its influence.

We simulated the motion of vesicles towards the membrane using Eq 15 with the same parameters as in Fig 7(A). Once at the membrane, each vesicle ‘waited a time *Δt*_*fus*_, obtained from the generated distribution of Fig 8(B), before producing a release event (spike).

**Fig 9:**
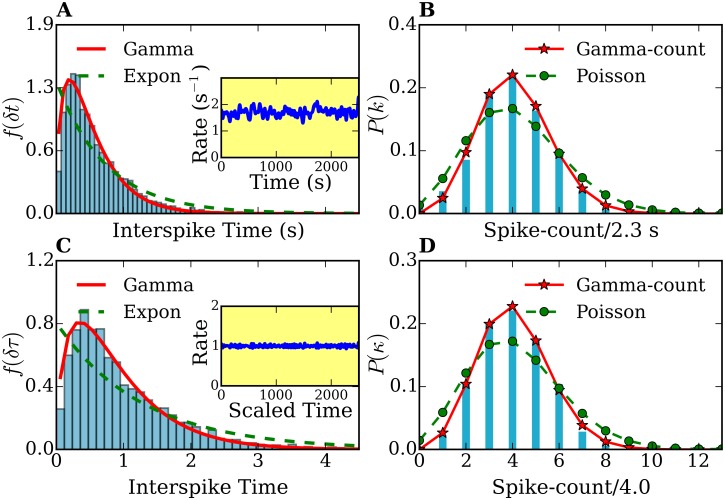
Interspike-time and frequency-count histograms computed from a simulation of diffusive vesicles under an attractive harmonic potential and including a lag time *Δt*_*fus*_ at the membrane (see text) according to Ref. [35], before and after time-rescaling. (A), (C) Interspike-time histograms *f*(*δt*) and *f*(*δτ*) before and after time rescaling, respectively. (B),(D) frequency-count histograms *P*(*k*) (for a time interval of *T* = 2.3 s width) and *P*(*κ*) (for a rescaled time interval of $\bar{\tau }=4.0$ width) before and after time rescaling, respectively. The histograms *f*(*δt*) and *f*(*δτ*) are fitted with gamma-(solid lines) and exponential distributions (dashed lines). The frequency-count histograms *P*(*k*) and *P*(*κ*) are fitted with the gamma-count (see text, solid lines) and Poisson distributions (dashed lines). The total number of spikes was 4257. Inset plots in (A) and (C) show the spike rates of the original and time rescaled spike series, respectively.

In Fig 9, we show a result of the simulations. As it can be seen by comparing Fig 9 with Fig 7, the lag between docking and fusion does not significantly affect the probability distribution of simulated exocytosis events, which can still be very well fitted by gamma processes. The histogram of Fig 9(A) is characterized by *μ* = 0.59 s and *σ* = 0.48 s. The parameters used for the fitting the histograms of 9 (A) are λ = 1.34 s^−1^, *p* = 1.62 and *θ* = 0.33 s. The histogram of Fig 9(C) is characterized by *μ*′ = 1.00 and *σ*′ = 0.8. The fitting parameters for the histograms 9(C) are λ′ = 0.78, *p*′ = 1.66 and *θ*′ = 0.56.

Notice that the mean of the interspike times histogram (*μ* = 0.59) does not change at all upon extension of the model to consider the fusion times. The standard deviation (*σ*) could show a tiny change ranging from 0.47 to 0.48 s in 15 simulations. This indicates that the priming/fusion process does not affect the interspike-time distributions. This fact is due to the difference in the time scales for priming/fusion and vesicle transport. According to the histogram of the lag times shown in Fig 8(B), the fusion times ranges between 10 and 250 milliseconds, and exhibits a main peak at 18 ms, which is 1–2 orders of magnitude smaller than the interspike times obtained in our experiments and our simulations of vesicle transport, which lie in the range of seconds. Taking this into account, the results of Fig 9 are not surprising.

**Fig 10:**
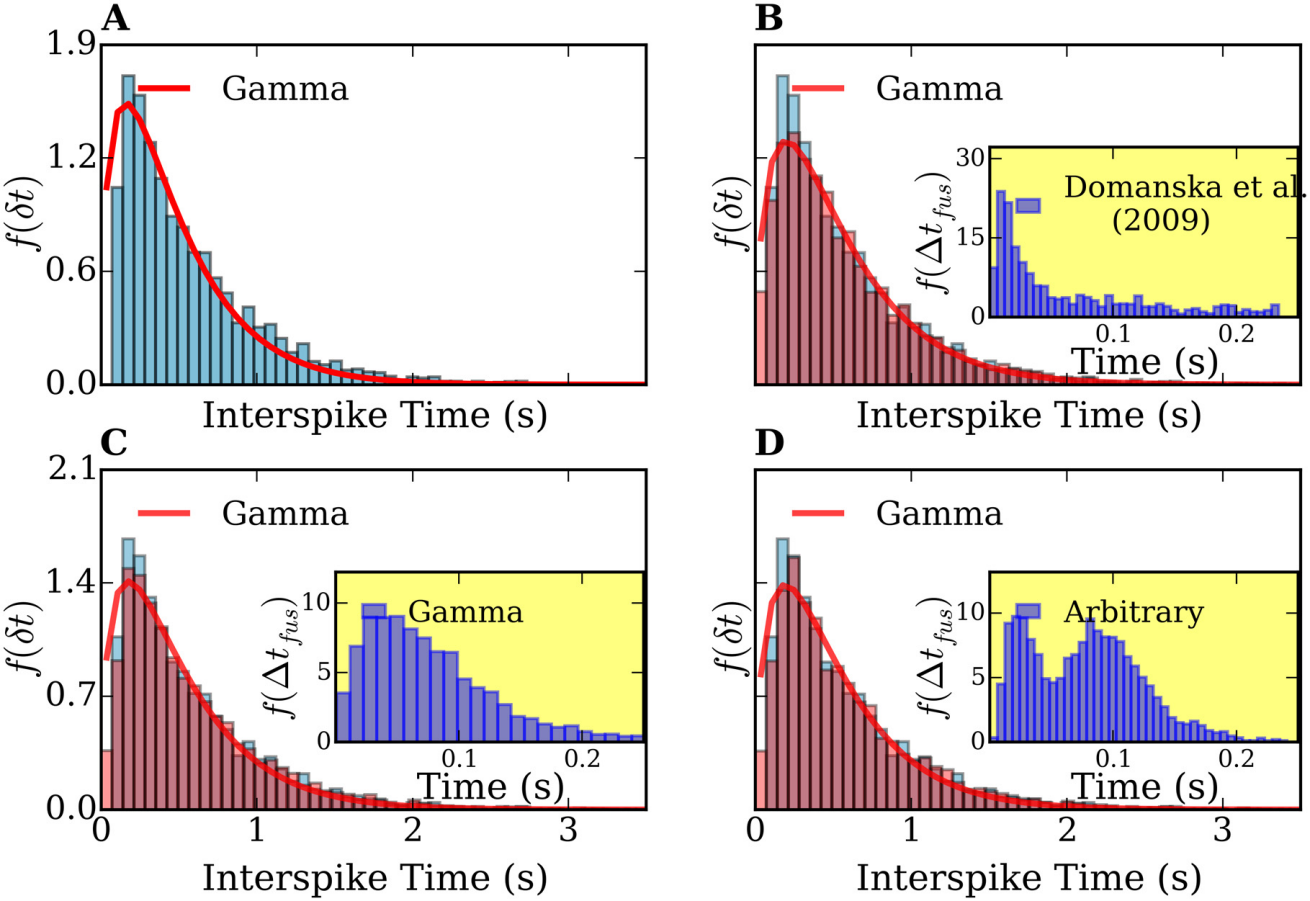
Comparison of interspike-time histograms computed from simulations of diffusive vesicles under an attractive harmonic potential and considering different fusion-time distributions. (A) Interspike-time histogram of a simulation without the fusion times, i.e., setting *Δt*_*fus*_ = 0. (B),(C),(D) show the histograms assuming *f*(*Δt*_*fus*_) from Ref. [35], a gamma distribution, and an arbitrary distribution, respectively. Inset plots in (B),(C),(D) show the assumed lag-time distributions.

Moreover, since the this difference in the time scales of the transport and fusion, the particular shape of *f*(*Δt*_*fus*_) is actually not relevant. To demonstrate this we performed simulations assuming different shapes for *f*(*Δt*_*fus*_). Results are shown in Fig 10. (A) shows the interspike-time histogram neglecting the influence of the lag times between docking and fusion, whereas Fig 10(B), 10(C) and 10(D) represent interspike-time histograms assigning the fusion times from Ref. [35], gamma shaped and an arbitrary function *f*(*Δt*_*fus*_), respectively. In all cases, the interspike-time histograms can be well fitted by gamma processes. Similar results were obtained assuming exponential-, Lévy-, generalized gamma- and Wald distributions of fusion times (not shown). For all the mathematically defined distributions *f*(*Δt*_*fus*_), we generated *Δt*_*fus*_ by using random generators from the scipy library [73].

From these results we conclude that the amperometric signals are governed by vesicle transport. This also means that the contribution of vesicles coming from the reserve pool is dominant in the amperometric signals, since our simulations only describe the transport of those vesicles.

## 7 Summary and Discussion

In this work, we proposed that it is possible to study vesicle transport in chromaffin cells by combining a numerical model based on Langevin simulations of vesicle motion and amperometric measurements of exocytosis in the *sustained regime*. We performed *ex-vivo* amperometric experiments on bovine chromaffin cells under Ba^2+^ stimulation to capture sustained exocytosis. The amperometric signals are, in principle, a superposition of releases from initially docked vesicles (docked pool) and from vesicles coming from the reserve pool. We performed a statistical analysis of the amperometric traces and modeled the spike sequences using point-processes. We were able to standardize the exocytosis activities of cells with different different activity rates by using the time-rescaling theorem. The statistics of the spike sequences from all performed measurements considerably deviates from that of a Poisson process and is reasonably well described by a two-parameter gamma distribution.

In order to explain this behavior we developed a model which relies on Langevin simulations of vesicle motion in 3 dimensions on the way to the membrane, described as a surface, at which release processes occur. We first simulated a purely diffusive ensemble of vesicles (Brownian motion) and showed that such a motion of vesicles leads to Poisson statistics of the corresponding spike series. Then, we considered an additional attractive harmonic potential towards the membrane in the Langevin equations of motion. We found that the corresponding simulated release statistics can be well fitted by a gamma distribution in very good agreement with our amperometric experiments. This indicates that there is a link between vesicle motion and catecholamines’ releases detected by amperometry. In order to study the influence of the priming/fusion processes on our numerical results we extended the model to include a lag time between docking and fusion of each vesicle using experimental results from the literature. We found that the priming/fusion reaction does not affect the interspike-time histograms. We propose that the gamma distribution is a signature of vesicle transport from the reserve pool to the active sites of the membrane. Therefore, the sequence of current peaks. This result suggests that the gamma distribution is a signature of vesicle transport from the reserve pool to the active sites of the membrane. Therefore, the sequence of current peaks from the amperometric signal (spike sequence) can contain essential information on vesicle motion.

From the comparison between experiment and simulations we then conclude that vesicles undergo lateral Brownian motion and at the same time direct motion towards the membrane prior catecholamines release. This result confirms and complements previous TIRFM evidences for active transport. The main point of this work is to extend this concept, gained from imaging studies on individual vesicles, to the whole ensemble of vesicles participating in exocytosis.

We note here that, apart from transport and priming/fusion mechanisms, other complex processes might also contribute to the amperometric signals. However; we assume that they do not significantly affect the characteristic of interspike series. For example, the ‘kiss and run’ type of releases should not affect our conclusions. As reported in Ref.[77], ‘kiss and run’ fusion processes are less frequent during prolonged stimulation, particularly when using Ba^2+^ as secretagogue. It is important to point out, that the attractive force towards the membrane that we assume in our simulations cannot *exactly* reproduce the amperometric spike statistics, but it makes the spike statistics deviate from a Poisson process in the same manner as the experimental signals. Although we could demonstrate that a correct description of vesicle motion requires an attractive force, we cannot give a conclusive statement regarding its shape. Other expressions for this force might also yield good agreement with experiments. Work in this direction is in progress. We expect our model to serve as a basis for further studies. In particular, it might be useful to confirm whether the model can also describe amperometric signals upon Ca^2+^ stimulation with the same degree of accuracy.

## Supporting Information

S1 File (PDF)
