# Supplementary material for: Vesicle Motion during Sustained Exocytosis in Chromaffin Cells: Numerical Model Based on Amperometric Measurements
Source: PLoS One. 2015 Dec 16;10(12):e0144045. doi: 10.1371/journal.pone.0144045 (PMC4699451; doi:10.1371/journal.pone.0144045)
Supplement: S1 File — (PDF) [file pone.0144045.s001.pdf]

S1 File

Vesicle Motion During Sustained Exocytosis in Chromaffin Cell: Numerical Model Based on Amperometric Measurements

Supporting Material

Daungruthai Jarukanont, Imelda Bonifás Arredondo, Ricardo Femat, Martin E. Garcia

S1.1 Data Acquisition and Spike Detection

Signals from exocytosis events are affected by noise in amperometric recordings. Therefore the data has to be cleaned up before the spikes can be extracted for statistical analysis. We are only interested in the spike frequency, which provides information about the vesicle release probability, and not on the shapes and amplitudes of the spikes. In our studies, a peak detection method was performed to extract spikes corresponding to excocytosis events, without taking care on the temporal shape and amplitude of the spikes [1]. The extracted spikes can be represented by a sequence of the form

$$S = \{S_1,\ S_2, \dots, S_n\}, \tag{1}$$

where $S_i = S(t_i)$ is the value of the signal measured at time $t = t_i$.

S1.2 Noise Reduction and Data smoothing

In order to suppress signal fluctuations produced by noise we use a generalization of the moving-average method, which consists in writing each signal point S_i^{smooth}as an average over a sufficiently large number of adjacent points as

$$S_i^{smooth} = (\omega_l S_{i-l} + \dots + \omega_0 S_i + \dots + \omega_{-l} S_{i+l})/(2l+1), \tag{2}$$

where the weights ω_l are, in principle, arbitrary, but decrease with $|l|$. In the present work we calculate the smoothed signal as a discrete convolution of the form

$$S_i^{smooth} = \sum_{l=-\infty}^{\infty} (\omega_l \, S_{i+l}), \tag{3}$$

with a bell shape Hanning window (discrete) function given by

$$\omega_l = 0.5 - 0.5\cos\left(\frac{2\pi l}{M-1}\right), \qquad \text{for } 0 < l \leq M-1$$
$$\omega_l = 0 \quad \text{otherwise.} \tag{4}$$

M is the number of points we consider in the output window. The signal is prepared by introducing periodic boundary conditions in order to minimize finite-size effects. Since the resolution of the experimental data was $t_{i+1} - t_i = 0.0025$ s, we used $M = 25$, i.e., we average the signals over a window width of 0.0625 s. It is important to mention that this procedure does not affect the position of the spikes.

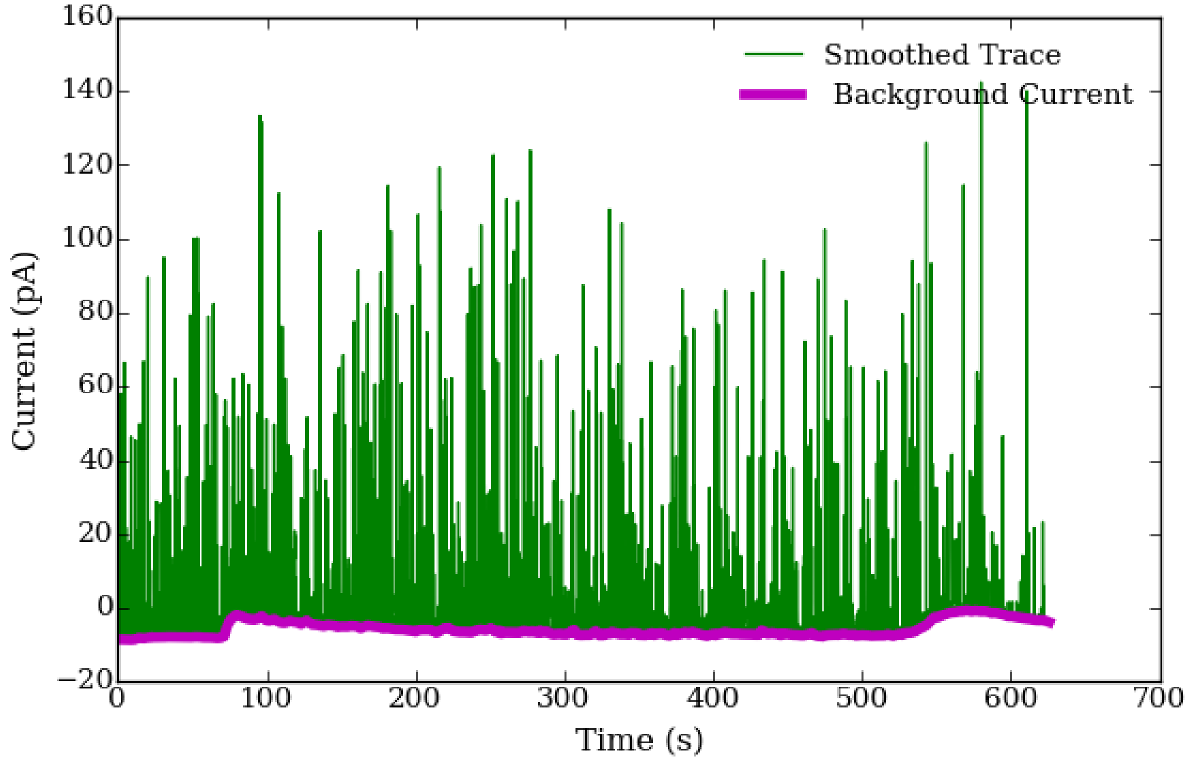

Figure S1: Smoothed signals of a particular cell with baseline estimate (thick line).

S1.3 Removal of background current

After smoothing the signal, we removed the minimum background current as the following.

(i) *Setting the minimum signal amplitude of the whole recording to zero.*

(ii) *Estimating the background current by dividing the data into small intervals of widths between* 0.5 *s and* 2.5 *s. Then, interpolating the baseline through the minima of all intervals.*

(iii) Removed the background current from the signal.

The application of the smoothing algorithm, and the base-line estimate is shown in Fig. S1.

S1.4 Peak Detection and Spike Selection

The positions of the peaks of current in the smoothed amperometric signal are obtained by calculating the numerical derivative of the signal and by identifying its zeros.

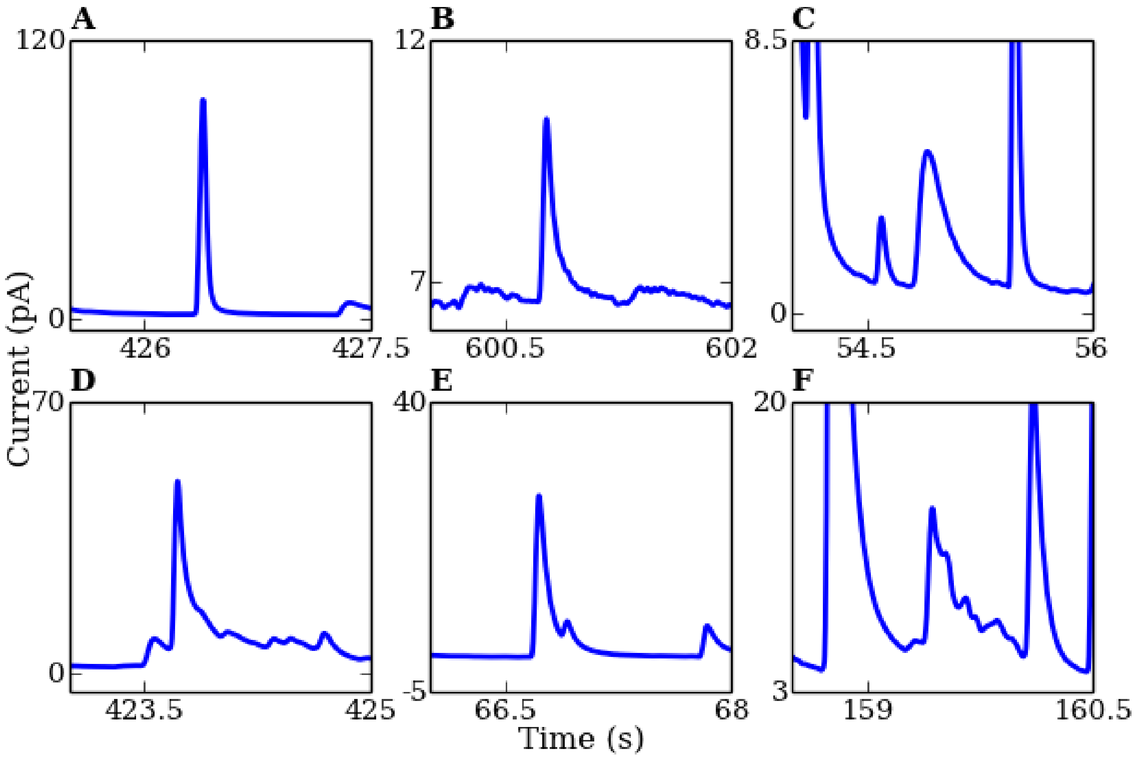

Figure S2: Peaks corresponding to different exocytosis events exhibiting distinct features. (A) Fully release event peak, (B) partial release event peak, (C) slow release event, (D) peak with pre sub-peak, (E) spike with post sub-peak, and (F) flickering peak.

Due to the complexity of the process of exocytosis, current peaks may exhibit different shapes, and not every zero of the signal-derivative corresponds to a full adrenaline release event. In Fig. S2 we show different examples of peaks with different shapes. A full release event is shown in Fig S2 (A). The current increases rapidly and then decreases exponentially. Fig S2 (B) shows a typical kiss-and-run process [2]. Events in which adrenaline release is slow

result in broad peaks of low intensity [Fig. S2 (C)]. Some peaks exhibit pre- and post sub-peaks, like those represented in Figs. S2 (D) and (E). Finally, Fig. S2 (F) contains an example of a flickering peak, corresponding to a stepwise adrenaline release.

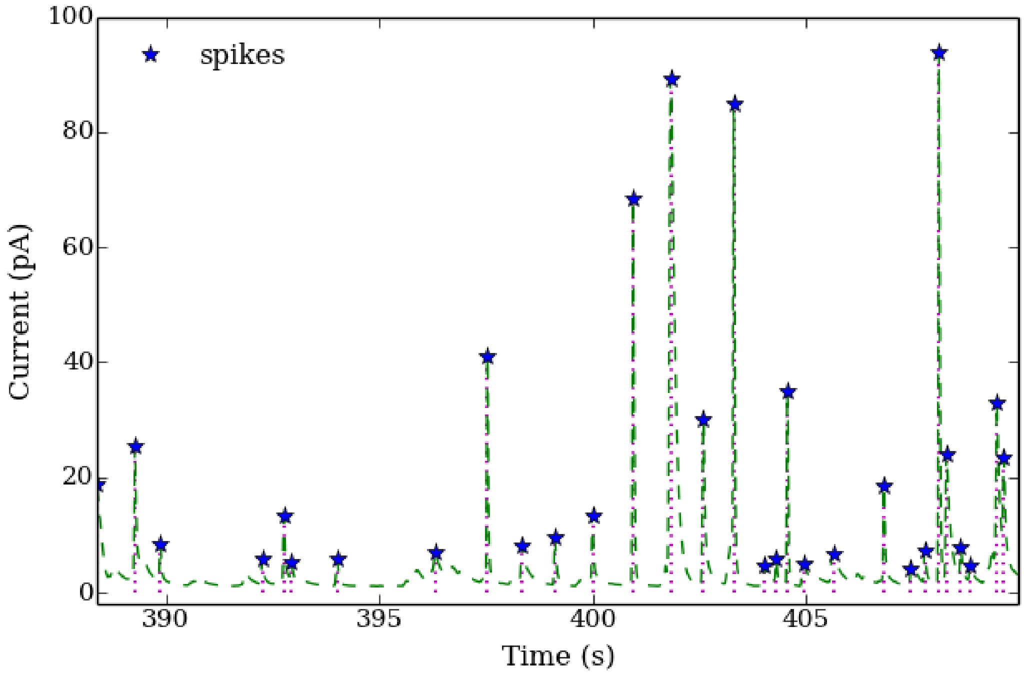

Figure S3: Series of spikes (stars) obtained from one amperometric recording by applying the criteria described in the text. The sequence of spikes as a time series is ready to be used for statistical analysis.

We use the following criteria to select current peaks which will be considered as spikes. We rule out:

(i) Peaks with signal smaller 3 times of rms noise of the baseline current (2-5 pA).

(ii) Peaks with low slope: These peaks may occur due to the superposition of the base of two close events, or due to fusion process far away from the scope. We filter out peaks with derivative smaller than a threshold value (25 %).

(iii) Pre- and post sub-peaks: since they belong to the main peak and do not correspond to additional events.

(iv) Small sub-peaks within a flickering peak, due to the same reason as in (iii).

Electrodes were held at +0.65 V with respect to a silver/silver chloride reference electrode using a modified picoamperometer (model AMU-130, Radiometer Analytical Instruments, Copenhagen, DK), for which the adjustable time-response was set at 50 μs. The output was digitalized at 40 kHz, displayed in real time and stored on a computer with no subsequent digital filtering.

Each amperometric trace obtained during cell secretion was visually and computationally inspected. By applying these criteria we reduce the noise and filter out superabundant peaks. Generally, 500 to 900 peaks could be isolated from each trace following these criteria. An example of the series of spikes remaining after noise reduction and spike selection is shown in Fig. S3.

References

[1] Oweiss, K. G. Statistical signal processing for neuroscience and neuro-technology. Academic Press. 2010.

[2] Südhof, T., C. The synaptic vesicle cycle. Annu. Rev. Neurosci. 2004; 27: 509 - 547.
